# Broad-Spectrum Antibiotics Deplete Bone Marrow Regulatory T Cells

**DOI:** 10.3390/cells10020277

**Published:** 2021-01-30

**Authors:** Hyojeong Han, Hannah Yan, Katherine Y. King

**Affiliations:** 1 Section of Hematology and Oncology, Department of Pediatrics, Baylor College of Medicine, Houston, TX 77030, USA; hyojeong.han@bcm.edu; 2 Program in Immunology, Graduate School of Biomedical Sciences, Baylor College of Medicine, Houston, TX 77030, USA; Hannah.yan@bcm.edu; 3 Section of Infectious Diseases, Department of Pediatrics, Baylor College of Medicine, Houston, TX 77030, USA

**Keywords:** bone marrow suppression, antibiotics, T regulatory cells, hematopoietic stem cell

## Abstract

Bone marrow suppression, including neutropenia, is a major adverse effect of prolonged antibiotic use that impairs the clinical care and outcomes of patients with serious infections. The mechanisms underlying antibiotic-mediated bone marrow suppression remain poorly understood, with initial evidence indicating that depletion of the intestinal microbiota is an important factor. Based on our earlier studies of blood and bone marrow changes in a mouse model of prolonged antibiotic administration, we studied whether changes in megakaryocytes or regulatory T cells (Tregs), two cell types that are critical in the maintenance of hematopoietic stem cells, contribute to antibiotic-mediated bone marrow suppression. Despite increased platelet numbers, megakaryocytes were unchanged in the bone marrow of antibiotic-treated mice; however, Tregs were found to be significantly depleted. Exogenous addition of Tregs was insufficient to rescue the function of bone marrow from antibiotic-treated mice in both colony formation and transplantation assays. These findings indicate that the intestinal microbiota support normal Treg development to protect healthy hematopoiesis, but that the restoration of Tregs alone is insufficient to restore normal bone marrow function.

## 1. Introduction

Antibiotic therapy is necessary to treat life-threatening bacterial infections, with approximately 249.8 million antibiotic prescriptions being issued in the United States annually [1]. However, prolonged use of antibiotics (two weeks or longer) is associated with adverse events, including bone marrow suppression [2,3,4,5]. With incidence ranging between 5–34%, bone marrow suppression is one of the most common antibiotic-associated adverse events, and can present as anemia and/or neutropenia [6]. Antibiotic-associated bone marrow suppression leads to unplanned outpatient visits, hospitalizations and early discontinuation of antibiotic therapy [3]. Unfortunately, survival for some, particularly immunocompromised oncology and bone marrow transplant patients, may depend on continuing antibiotic therapy, precluding early discontinuation. Despite the magnitude of this problem, the mechanisms underlying antibiotic-associated bone marrow suppression remain poorly understood.

Our lab demonstrated that prolonged antibiotic use suppresses hematopoiesis not through direct inhibition of hematopoietic stem cells (HSCs), the progenitors of all blood cells, but instead indirectly, through depletion of the intestinal microbiome [6,7]. Antibiotics do not exert bone marrow suppressive effects on germ free mice, and restoration of peripheral blood counts in antibiotic-treated mice can be hastened by reestablishing the bacterial intestinal microbiome [7]. In addition, our studies revealed that antibiotic-treated mice display thrombocytosis and have a decreased CD4:CD8 ratio in bone marrow [7]. However, the exact mechanisms by which antibiotics suppress bone marrow function remain an open question. 

The bone marrow niche surrounding HSCs is composed of many different cell types, including stromal cells and mesenchymal stem cells, and is known to regulate HSC proliferation, quiescence, self-renewal and differentiation [8]. Megakaryocytes and regulatory T cells in the bone marrow niche regulate hematopoiesis by promoting HSC quiescence [9,10] and providing an immune niche to evade host immunity [11,12,13] and favor HSC survival, respectively [14]. Based on these known roles, and the changes in platelets and in the CD4:CD8 ratio observed in our prior experiments, we hypothesized that megakaryocytes and Tregs might play a role in antibiotic-associated bone marrow suppression. Here, we use flow cytometry, colony-formation and transplant assays and genetic mouse models to test the hypotheses that expansion of megakaryocytes promotes bone marrow suppression by enhancing HSC quiescence, while depletion of Tregs promotes bone marrow suppression through HSC destruction during antibiotic therapy.

## 2. Materials and Methods

### 2.1. Mice

We used 6- to 14-week-old CD45.1 or CD45.2 C57BL/6 male and female mice for all experiments. We obtained B6.129(Cg)-Foxp3tm(DTR/GFP)Ayr/J stock #016958 (Foxp3 conditional knockout; Foxp3-DTR) mice from The Jackson Laboratory. All mice were bred and housed in specific pathogen-free (SPF) conditions in the animal facility at Baylor College of Medicine (BCM) in accordance with a protocol approved by the Institutional Animal Care and Use Committee. 

### 2.2. Antibiotic Treatment

Mice were fed water ad libitum containing antibiotics: 1 g/L ampicillin, 0.5 g/L vancomycin, 1 g/L metronidazole, and 1 g/L neomycin (all from Sigma, St. Louis, MO, USA) based on a prior study (Josefsdottir et al., 2017). We added flavoring (20 g/L grape-flavored sugar-free Kool-Aid Drink Mix [Kraft Foods Global, Inc., Chicago, IL, USA]) to the drinking water for all groups. Mice were treated for 14 days. 

### 2.3. Blood and Bone Marrow Analysis

Whole blood was collected either by cardiac puncture or retro-orbital sinus puncture. We mixed whole blood with EDTA (final concentration 5mM) and analyzed complete blood counts using an Advia 120 Hematology system (Siemens, Munich, Germany). 

Peripheral blood mononuclear cells (PBMCs) were isolated from whole blood by standard methods. Bone marrow was obtained by flushing from femur or tibia, and cells were counted using Cellometer^®^ Auto 2000 (Nexcelom Bioscience, Lawrence, MA, USA) at 1:1 dilution in ViaStain^TM^ AOPI staining solution (Nexcelom Bioscience). Red blood cells were lysed using red blood cell lysis buffer. Cells were stained with antibody mixes at concentration 1:100 for each surface marker antibody in Hank’s buffered saline solution with 2% fetal bovine serum and 1% HEPES buffer. For Foxp3 staining, cells were stained with surface marker antibodies, fixed using Fixation/Permeabilization (Invitrogen, Carlsbad, CA, USA) diluted in eBioscience^TM^ Fixation/Perm Diluent (Invitrogen), and stained with Foxp3 antibody at 1:100 concentration in 1× permeabilization buffer (Invitrogen). Multicolor flow cytometry was performed on LRSFortessa cell analyzer (BD Biosciences, San Jose, CA, USA) or LSRII cell analyzer (BD Biosciences), and flow data were analyzed using FlowJo (FlowJo, LLC, Ashland, OR, USA). 

### 2.4. Thymus Analysis

Harvested thymi were crushed and filtered through a 40 μM cell strainer. Cells were counted using Cellometer^®^ Auto 2000 (Nexcelom Bioscience) at 1:1 dilution in ViaStain^TM^ AOPI staining solution (Nexcelom Bioscience). Red blood cells were lysed using red blood cell lysis buffer. Cells were fixed, stained, and analyzed as described for the bone marrow.

### 2.5. CD25 Depletion/Enrichment from Bone Marrow

Bone marrow or spleen from CD45.2 or CD45.1 mice was stained with CD25-biotin antibody (Invitrogen) and then incubated with anti-biotin microbeads (Miltenyi Biotec, Bergisch Gladbach, Germany). An autoMACS^®^ Pro Separator (Miltenyi Biotec) was used to separate CD25-depleted and CD25-enriched samples.

### 2.6. Methylcellulose Culture

Pooled whole bone marrow from antibiotic-treated or control mice (CD45.2) was cultured in methylcellulose with appropriate murine cytokines (MethoCult^TM^ GFM3434, Stemcell technologies) with either depletion or addition of CD25 cells (see CD25 depletion/enrichment methods). A total of 20,000 whole bone marrow cells were plated per well in six-well tissue culture plates incubated at 37 degrees, 5% CO_2_, and colonies were counted after five days. 

### 2.7. Bone Marrow Transplant

We obtained bone marrow by crushing the leg bones from CD45.2 or CD45.1 mice using a mortar and pestle and suspending cells in Hank’s Buffered Saline Solution with 1% penicillin/streptomycin (Gibco, Waltham, MA, USA) and 1% HEPES. Bone marrow from antibiotic-treated or control mice were pooled and counted. Pooled bone marrow cells were further divided into six groups as follows: control whole bone marrow (WBM), control WBM depleted of CD25+ cells, or control WBM with addition of CD25+ cells, WBM from antibiotic-treated mice, or WBM from antibiotic-treated mice with depletion or addition of CD25+ cells. Two days prior to transplant, Foxp3-DTR mice received 50 μg/kg of diphtheria toxin (Sigma) via intraperitoneal injection every day for two days. Mice received 10.5 Gy irradiation in 2 doses split 4 h apart and were transplanted immediately thereafter. For each group, 2 × 10^5^ donor WBM cells were combined with 2 × 10^5^ cells of competitor marrow (CD45.1), and this combination was injected into irradiated Foxp3-DTR recipients. For the two groups with added CD25+ cells, each Foxp3-DTR recipient mouse received 1 × 10^6^ CD25+ cells, which was added to the mixed bone marrow prior to injection. We assessed engraftment every 4 weeks for up to 16 weeks from whole blood obtained via retro-orbital bleed and then performed flow cytometry for lineage composition. Whole bone marrow was assessed at 16 weeks for hematopoietic stem and progenitor cells (HSPC), neutrophils and regulatory T cells. 

## 3. Results

### 3.1. Antibiotic Treatment Does Not Suppress Bone Marrow through Increased Megakaryocyte Progenitor Counts or Altered Megakaryocyte Morphology 

**Figure 1:**
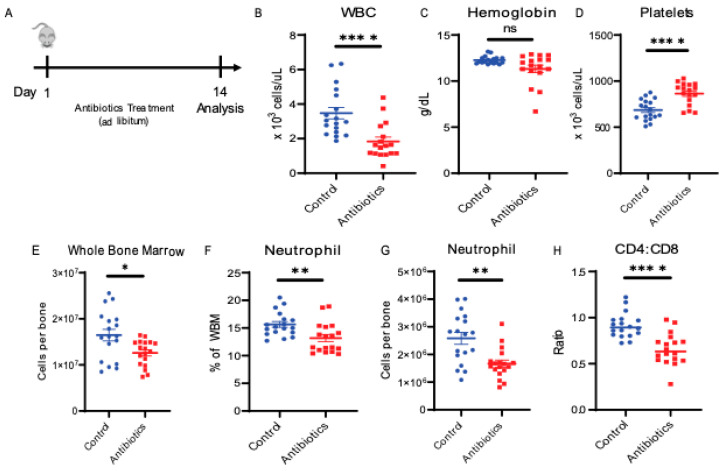
Antibiotic administration alters peripheral blood and bone marrow cell composition. (**A**) Antibiotic treatment experiment design. (**B**) White blood cell counts (WBC), (**C**) hemoglobin, (**D**) platelet counts from peripheral blood using automated hematologic cell counter and compared between control vs. antibiotic-treated (antibiotics). (**E**) Whole bone marrow (WBM) cells comparing control vs. antibiotics were counted using automated cell counter. (**F**,**G**) Neutrophil population from WBM for control vs. antibiotics are shown using flow cytometry. (**H**) CD4:CD8 ratio in WBM for control vs. antibiotics are shown using flow cytometry. Results are compiled from 3 independent experiments (*n* = 5–8 per experiment). Graphs show mean + SEM. Statistical significance was determined by Mann–Whitney U test. ns, not significant, * *p* < 0.05, ** *p* < 0.01, **** *p* < 0.0001.

To discover the mechanism through which antibiotics induce bone marrow suppression, we first treated mice with a four-antibiotic cocktail of vancomycin, neomycin, ampicillin and metronidazole (VNAM) in the drinking water for two weeks (Figure 1A). As seen in our previous work [7], antibiotic-treated mice became leukopenic compared to the control mice (Figure 1B). We did not observe anemia (Figure 1C), but the antibiotic-treated mice consistently demonstrated a significant degree of thrombocytosis (Figure 1D). We performed flow cytometric analysis of bone marrow to identify differentiated cell populations (Supplementary Figure S1). These analyses revealed that antibiotic-treated mice demonstrated decreased neutrophil count, CD4:CD8 ratio and bone marrow cellularity (Figure 1E–H) compared to the control mice. Our previous work has demonstrated that the decrease in CD4:CD8 ratio is due to a decrease in CD4 T cells with no change in CD8 T cells [7]. Thus, as expected, antibiotic therapy suppresses bone marrow, resulting in a decreased CD4:CD8 ratio, neutropenia and leukopenia [7].

**Figure 2:**
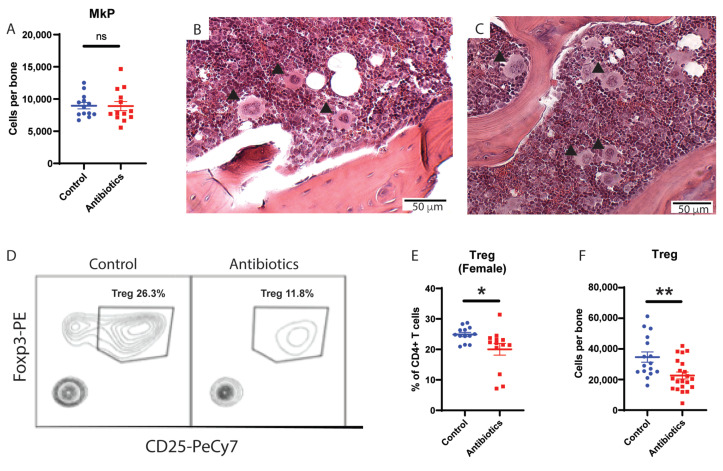
Antibiotic administration does not change megakaryocyte progenitors (MkP) but leads to decrease in regulatory T cells (Treg) in bone marrow. (**A**) MkP population from whole bone marrow (WBM) for control vs antibiotic-treated (antibiotics) are shown using flow cytometry. MkP (solid arrows) size, shape and number from bone marrow are compared between (**B**) control and (**C**) antibiotics (hematoxylin and eosin staining of bone marrow from femur, 10×). (**D**) Flow cytometry plot of Treg population comparing control and antibiotics-treated group. (**E**) Treg population from WBM of female mice for control vs antibiotics. (**F**) Number of Treg per tibia for male and female control vs antibiotics-treated mice are shown. Results are compiled from 2–3 independent experiments (*n* = 5–8 per experiment). Graphs show mean + SEM. Graphs show mean + SEM. Statistical significance was determined by Mann-Whitney U test. ns, not significant, * *p* < 0.05, ** *p* < 0.01.

To better understand the etiology of antibiotic-associated bone marrow suppression, we evaluated the number of megakaryocytes and megakaryocyte progenitors in the bone marrow by flow cytometry (Supplementary Figure S2A). Despite increased platelet counts, antibiotic-treated mice did not have increased megakaryocyte progenitors compared to control mice (Figure 2A). Furthermore, megakaryocytes from the antibiotic-treated mice were of similar size and morphology compared to controls (Figure 2B,C). These data indicate antibiotic treatment does not significantly alter megakaryocytes in mice.

### 3.2. Antibiotic Treatment Depletes Regulatory T Cells in Murine Bone Marrow

As Tregs create an immunoprotective environment that is important for supporting HSC survival and function [11,12,13], we next measured regulatory T cells (Tregs) in the bone marrow of antibiotic-treated mice compared to controls. Tregs were identified as CD4+ FoxP3+ CD25+ cells in the bone marrow by flow cytometry (Supplementary Figure S2B). Antibiotic-treated mice had a significant (~2-fold) drop in Treg cells compared to controls (Figure 2D). Since baseline numbers of Tregs differ in male and female mice, with males having higher baseline number of Tregs as a percentage of all T cells, we measured Tregs in both groups separately (Figure 2E [female] and Supplementary Figure S2F [male]). Of note, we found a significant drop in Tregs in the bone marrow of both males and females compared to controls (Figure 2F). Consistent with this change, the size of the thymus, the total number of cells per thymus, and the number of Tregs per thymus all decreased in antibiotic-treated mice (Supplementary Figure S2C–F). These findings indicate that antibiotic treatment has a negative effect on Tregs in the bone marrow. 

## 3.3. Tregs Are Insufficient to Rescue WBM Cell Counts and Engraftment in Antibiotic-Treated Mice

**Figure 3:**
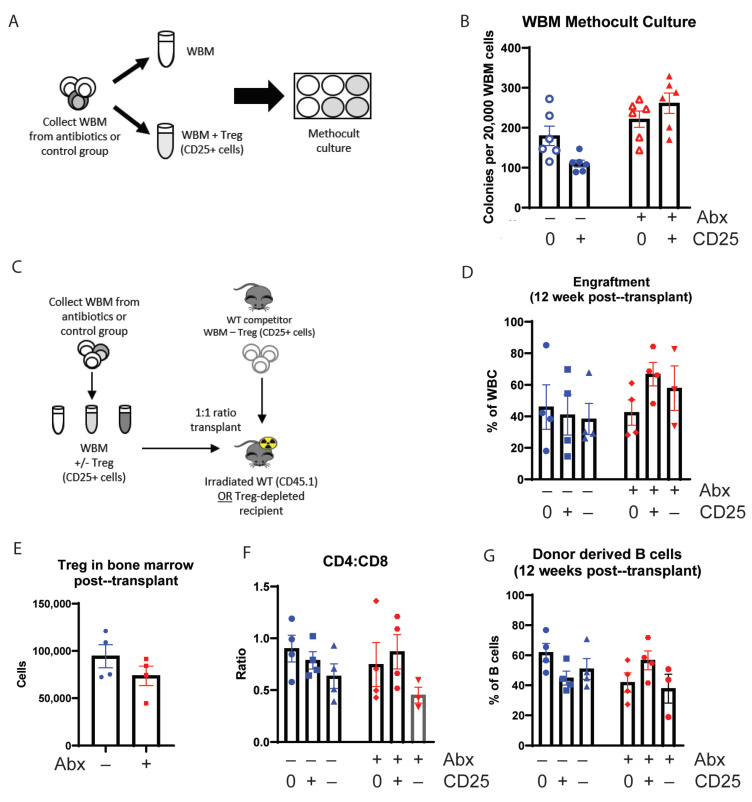
Addition of Treg (CD25+ cells) to antibiotic-treated mice whole bone marrow (WBM) improve cell count in vitro and engraftment in vivo. (**A**) Methocult culture experiment design (**B**) Colonies from WBM cells were counted in six replicates on day 5 for each group. Results are compiled from 1 independent experiment, *n* = 2–3. (**C**) Bone marrow transplant (BMT) experiment design using Treg-depleted recipient mice (**D**) Engraftment at 12 weeks after transplant analyzed from WBC from donor cells using flow cytometry. (**E**) Treg population from WBM at 16 weeks after transplant using flow cytometry. (**F**) CD4:CD8 ratio from WBM at 16 weeks after transplant. (**G**) B cell contribution from donor-derived WBC at 12 weeks after transplant. N = 3–5. Graph show mean + SEM. No statistical differences by Kruskal-Wallace.

Next, we investigated whether exogenously adding Tregs back to bone marrow from antibiotic-treated mice could rescue its function. First, we added Tregs (CD25+ cells) to WBM harvested from control or antibiotic-treated mice and assessed colony formation capacity in methylcellulose (Figure 3A). Consistent with a total loss of cellularity and relative enrichment in progenitors, we observed a slightly higher but statistically insignificant baseline rate of colony formation from the antibiotic-treated group. Adding Tregs did not lead to a statistically significant change in colony-forming capacity for either the control or antibiotic-treated marrow (Figure 3B). These results suggest that repletion of Tregs to marrow of antibiotic-treated mice is insufficient to restore the colony-forming ability of hematopoietic progenitors.

To determine if the addition of Tregs could alter bone marrow function in vivo, we either depleted or added CD25+ Treg cells to harvested WBM from control or antibiotic-treated mice. Then, we transplanted the cells to lethally irradiated Foxp3-DTR mice, in which endogenous Tregs had been depleted by diphtheria toxin administration (Figure 3C). Addition of Tregs to control marrow had no effect on total engraftment (Figure 3D), and we did not observe any significant changes in peripheral blood and WBM counts (Supplementary Figure S3A–D). We did note a trend toward improved engraftment in mice that received Treg-supplemented WBM from antibiotic-treated mice compared to those that received WBM from antibiotic-treated mice alone (Figure 3D, Supplementary Figure S3E); however, these differences did not reach statistical significance.

Notably, Treg counts remained low 16 weeks after transplant in mice transplanted with WBM from antibiotic-treated mice compared to recipients of control WBM, even though these recipients were never treated with antibiotics themselves (Figure 3E). The CD4:CD8 ratio was not impacted by the addition of Tregs (Figure 3F). Whereas adding Tregs to control WBM did not change the B cell engraftment in mice transplanted with control WBM, we found that recipients of WBM from antibiotic-treated mice had a statistically insignificant tendency toward decreased B cell counts, and the addition of Tregs tended to increase B cell counts in mice transplanted with WBM from antibiotic-treated mice (Figure 3G, Supplementary Figure S3F–G). These findings are consistent with the known role for Tregs in B cell development [15].

## 4. Discussion

In this study, we investigated two aspects of the bone marrow microenvironment that could contribute to antibiotic-associated bone marrow suppression [8]. Since megakaryocytes have been shown to regulate hematopoiesis by favoring HSC quiescence [9,10], we assessed how antibiotic treatment affects megakaryocytes, but found no differences in megakaryocyte count, size or morphology following therapy. Next, because Tregs support an immunoprotective environment that promotes HSC survival [11,12,13], we evaluated their presence in mouse bone marrow following prolonged antibiotic therapy. We found that antibiotic treatment significantly decreased Treg numbers in the bone marrow compared to control mice. However, the addition of Tregs (CD25+ cells) to the WBM of antibiotic-treated mice was insufficient to rescue either colony formation in methylcellulose culture or engraftment in Treg-deficient FTR mice. Taken together, our findings suggest that loss of Tregs may be a contributor to bone marrow suppression in the setting of prolonged antibiotic administration, but that restoration of Tregs alone is insufficient to restore normal hematopoiesis.

Our investigation into megakaryocytes was motivated by a prior observation that platelet counts are consistently and significantly increased in antibiotic-treated mice compared to control mice [7]. Since megakaryocytes promote HSC quiescence [9,10], we hypothesized that antibiotic treatment may increase megakaryocytes and thus enforce excessive HSC quiescence to contribute to bone marrow suppression. However, we found no difference in megakaryocyte counts and morphology between antibiotic-treated and control mice, indicating that alterations in megakaryocytes do not cause antibiotic-mediated bone marrow suppression. As platelets are an acute phase reactant, their abundance in antibiotic-treated mice may reflect the increased intestinal and systemic inflammation that results from depletion of normal microbiota or loss of Tregs, as described in this report.

In contrast, we found that Tregs were depleted from the bone marrow of antibiotic-treated mice. Tregs have been previously implicated in healthy bone marrow function. For example, activation of cytotoxic T cells and decreased Treg counts are believed to be the etiology of idiopathic severe aplastic anemia, a bone marrow failure disorder [16]. While there were some trends in the data regarding the effects of the exogenous addition of Tregs to marrow of antibiotic-treated mice, we were unable to demonstrate improved methylcellulose colony formation or engraftment of WBM into a Treg-deficient mouse background. This lack of efficacy likely indicates that there are other factors at play in mediating antibiotic-associated bone marrow suppression. Furthermore, we did not validate that all the CD25+ cells were bona fide Tregs, nor that they efficiently reached sites of critical function in our experiments. We also observed that WBM from antibiotic-treated mice produced fewer Tregs and a lower CD4:CD8 ratio after transplant compared to controls, even though the transplant recipients were not treated with antibiotics. Thus, changes in T cell and Treg production after antibiotic treatment were persistent even after transplant, indicating the environment may sustainably influence the epigenetic control of their production. Finally, we noted that adding Tregs to WBM from antibiotic-treated mice tended to improve B cell counts compared to WBM from antibiotic-treated mice alone. Our findings support those in a prior report by Pierini et al., that demonstrated that Tregs play an important role in B cell lymphopoiesis and are necessary for B cell differentiation [15].

Our study has several limitations. We did not examine all the possible cell types in the bone marrow that play important roles in regulating hematopoiesis. For example, mesenchymal stem cells (MSCs) are a key cell type involved in the maintenance of HSCs, and patients with severe aplastic anemia have an increased rate of MSC apoptosis. A prior study showed that coculturing peripheral blood mononuclear cells (PBMCs) with MSCs from patients with aplastic anemia reduced PBMC proliferation compared to MSCs from healthy patients [17]. Future studies will be needed to assess the potential role of MSCs in the etiology of antibiotic-associated bone marrow suppression. In addition, components or products of the intestinal microbiome may signal directly to the bone marrow to maintain normal function, and depletion of these products by antibiotic administration may be a key contributor to antibiotic-associated bone marrow suppression.

In summary, our studies demonstrate that Tregs are significantly depleted in the setting of prolonged broad-spectrum antibiotic treatment, and may contribute to antibiotic-associated bone marrow suppression. These findings are consistent with the known role of the microbiome in Treg development and function [18]. However the exogenous addition of Tregs was insufficient to rescue bone marrow suppression, indicating that additional factors contribute. Further work to gain a mechanistic understanding of the etiology underlying antibiotic-associated bone marrow suppression will be needed to develop new strategies to prevent adverse effects of antibiotics and enable optimal antibiotic management, particularly in vulnerable oncology and BMT patients.

## Data Availability

Not applicable.

## Supplementary Materials

The following are available online at https://www.mdpi.com/2073-4409/10/2/277/s1, Figure S1: Flow cytometry plot for neutrophils and hematopoietic stem and progenitor cells (HSPC) from bone marrow. Figure S2: Regulatory T cells (Treg) are also decreased in thymus in antibiotic-treated (antibiotics) mice along with thymus weight and cell count. Figure S3: Addition of CD25+ cells show improved engraftment and B cell distribution trend but complete blood count (CBC) and whole bone marrow (WBM) counts do not change.
