# Supplementary material for: Broad-Spectrum Antibiotics Deplete Bone Marrow Regulatory T Cells
Source: Cells. 2021 Jan 30;10(2):277. doi: 10.3390/cells10020277 (PMC7911786; doi:10.3390/cells10020277)
Supplement: Supplementary file 1 [file cells-10-00277-s001.pdf]

Supplementary Figures

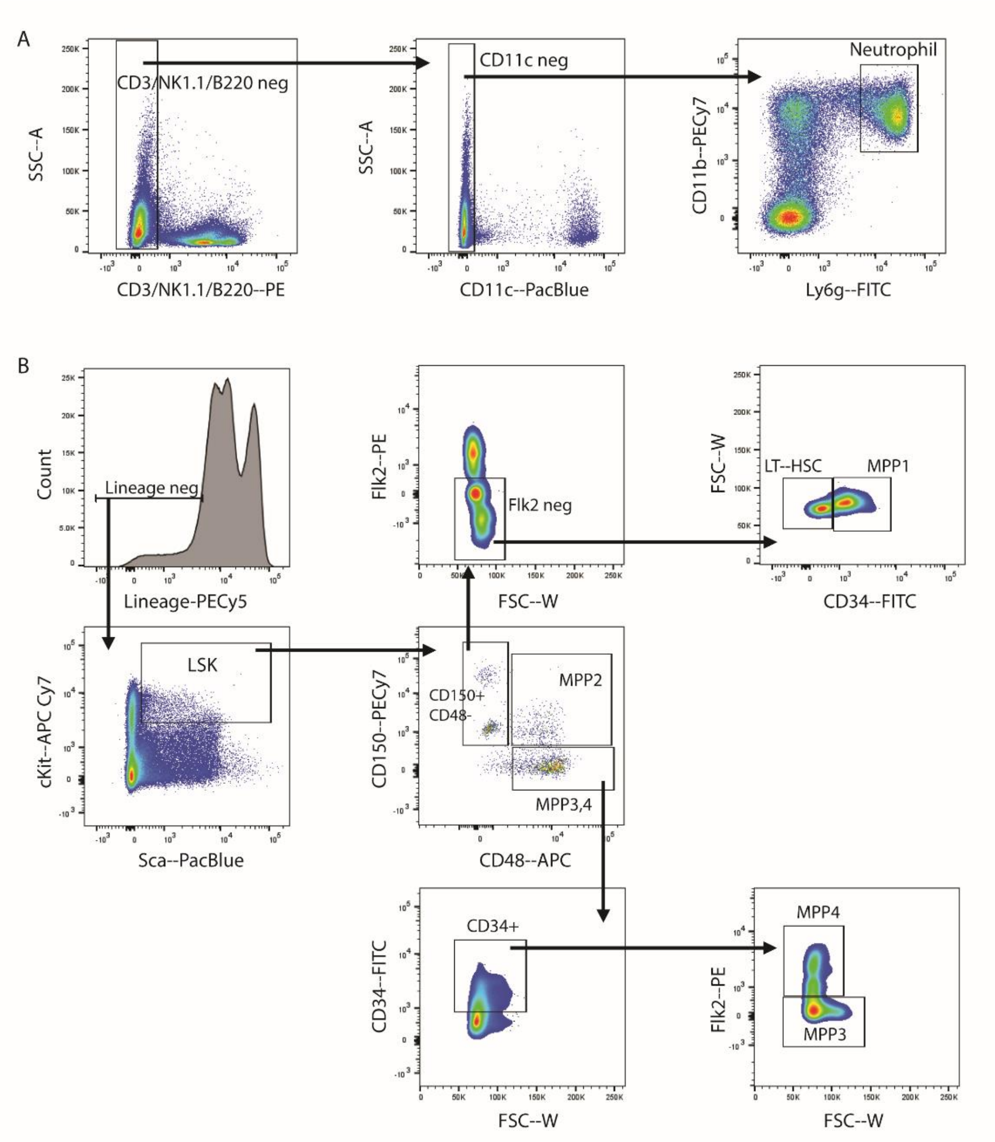

Figure S1: Flow cytometry plot for neutrophils and hematopoietic stem and progenitor cells (HSPC) from bone marrow. (**A**) After exclusion of cell debris using forward-scatter (FSC) and side-scatter (SSC) and dead cells using DAPI, myeloid cells were selected by excluding B cells (B220 stained), T cells (CD3 stained), natural killer cells (NK1.1), dendritic cells (CD11c stained). Neutrophils were selected (CD11b and Ly6g stained). (**B**) After exclusion of cell debris using FSC and SSC and dead cells using DAPI, lineage positive cells (Gr1, CD11b, B220, CD4, CD8 and Ter119 stained) were excluded. cKit against Sca-1 (Sca) were used to identify LSK cells (cKit and Sca-1 stained). LSK brought forward to CD150 against CD48 to identify multipotent progenitor 2 (MPP2) (CD150 and CD48 stained). CD150+CD48- population was brought forward to identify long-term hematopoietic stem cells (LT-HSC) (Flk2-CD34-) and MPP1 (Flk2-CD34+). CD150-CD48+ (MPP3,4) population was brought forward to identify MPP3 (CD34+Flk2-) and MPP4 (CD34+Flk2+).

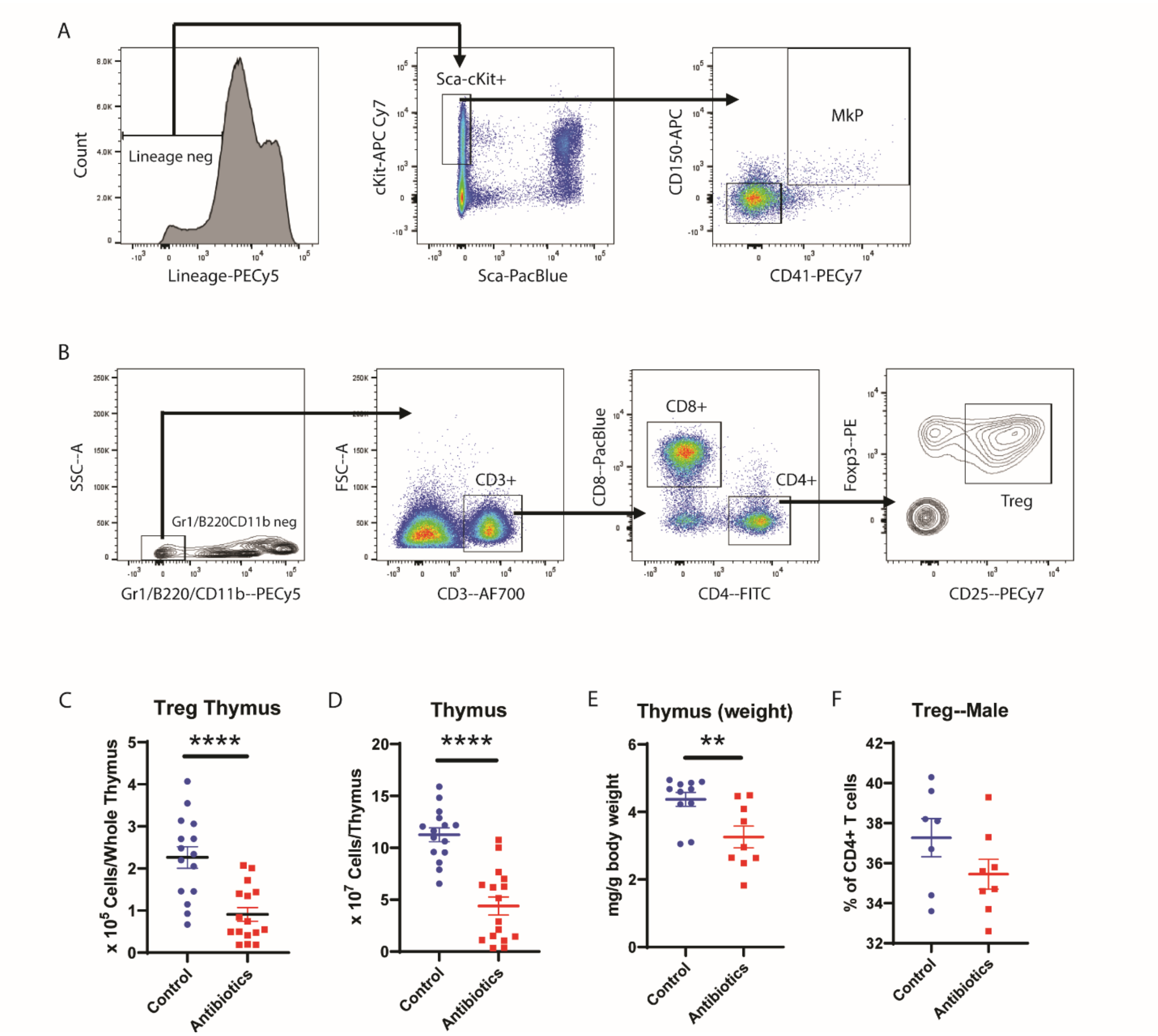

Figure S2: Regulatory T cells (Treg) are also decreased in thymus in antibiotic-treated (antibiotics) mice along with thymus weight and cell count. (**A**) Representative flow plot for bone marrow MkP. After exclusion of cell debris using FSC and SSC and dead cells using DAPI, lineage positive cells (Gr1, CD11b, B220, CD4, CD8 and Ter119 stained) were excluded. cKit against Sca-1 (Sca) were used to identify cKit$^+$Sca-1$^-$ cells. MkP populations (CD150$^+$ and CD41$^+$) were selected. (**B**) Representative flow plot for Tregs. After excluding cell debris using forward-scatter (FSC) and side-scatter (SSC), and selecting single cells, T cells (CD3+) were selected by excluding B cells (B220,) and myeloid cells (Gr1, CD11b). Tregs were selected from CD4+ stained (CD4+ T cells) with Foxp3 and CD25 stained cells. (**C**) Treg population from thymus comparing control vs antibiotics-treated. (**D**) Thymus cell count for control vs antibiotics. (**E**) Thymus weight (mg) per body weight (g) is shown. (**F**) Treg population from male mice from bone marrow Results are compiled from 3 independent experiments (n = 5–8 per experiment). Graphs show mean ± SEM. Statistical significance was determined by Mann-Whitney U test. ns, not significant, $^{**}P < 0.01$, $^{****}P < 0.0001$.

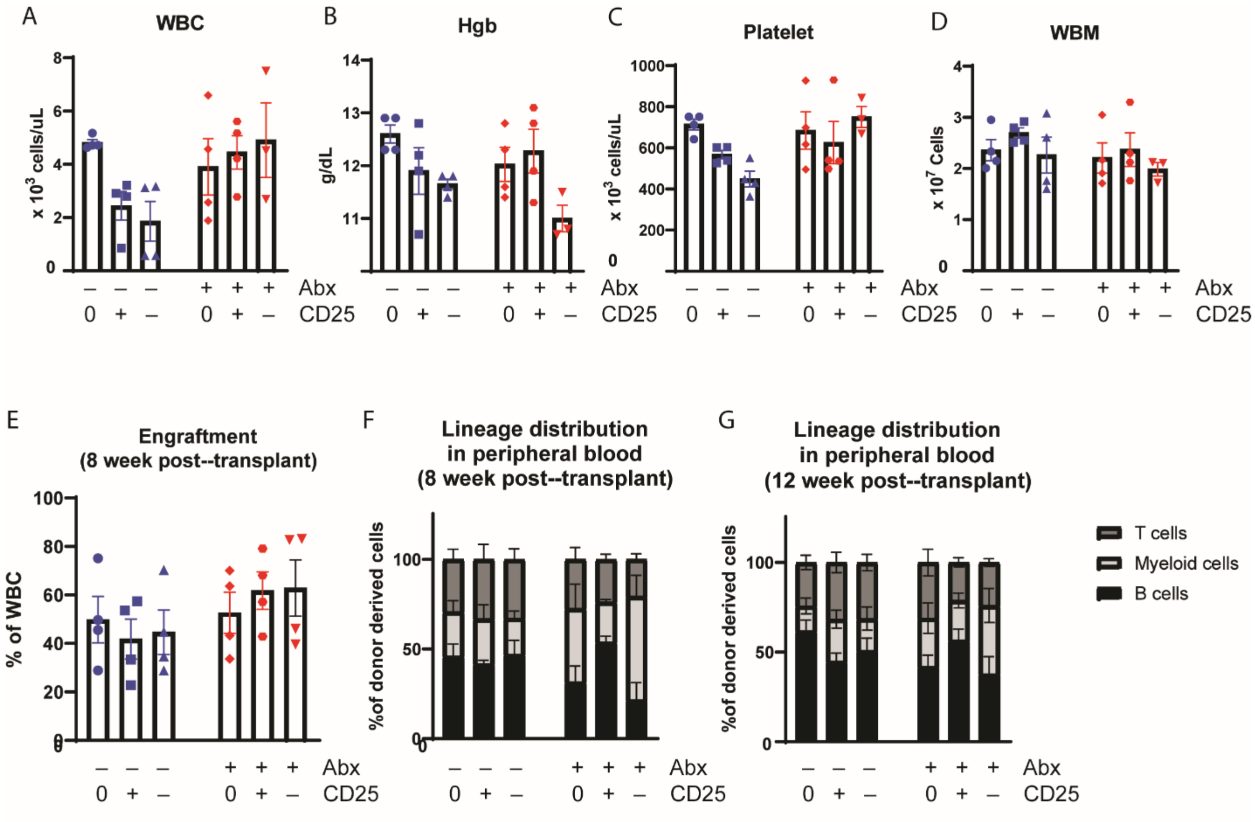

Figure S3: Addition of CD25+ cells show improved engraftment and B cell distribution trend but complete blood count (CBC) and whole bone marrow (WBM) counts do not change. (**A**) WBC, (**B**) Hgb, (**C**) platelets from peripheral blood and (**D**) WBM cell counts of recipient mice at 16 week after transplant. Engraftment of donor cells in the peripheral blood of Foxp3--depleted mice recipient (**E**) at 8 weeks after transplant. Lineage distribution of donor cells in the peripheral blood of Foxp3-depleted recipient mice (**F**) at 8 weeks after transplant and (**G**) at 12 weeks after transplant. *n* = 3–5 per group. Graphs show mean ± SEM.
